# Injury Patterns and Incidence in an Elite Youth Football Academy—A Prospective Cohort Study of 138 Male Athletes

**DOI:** 10.3390/jcm12196138

**Published:** 2023-09-22

**Authors:** Johannes Weishorn, Ayham Jaber, Severin Zietzschmann, Jan Spielmann, Tobias Renkawitz, Yannic Bangert

**Affiliations:** 1 Department of Orthopaedics, Heidelberg University Hospital, Schlierbacher Landstrasse 200a, 69118 Heidelberg, Germany; johannes.weishorn@med.uni-heidelberg.de (J.W.); ayham.jaber@med.uni-heidelberg.de (A.J.); severin.zietzschmann@med.uni-heidelberg.de (S.Z.); tobias.renkawitz@med.uni-heidelberg.de (T.R.); 2 TSG ResearchLab gGmbH, Horrenberger Straße 58, 74939 Zuzenhausen, Germany; jan.spielmann@tsg-researchlab.de

**Keywords:** injury patterns, football, youth academy, soccer, sports injuries, elite football

## Abstract

Background: There is a lack of evidence regarding injury incidence in German elite youth football academies, and the risk of re-injury is unknown. Therefore, the objectives of this study were (1) to determine injury patterns and incidence in an elite youth football academy in Germany, (2) to monitor overuse-/trauma-related injuries over the course of the season, and (3) determine the risk of re-injury. Methods: A prospective cohort study was conducted in the 2012/2013 season among 138 male players from an elite youth football academy in Germany. Injuries were recorded according to the consensus statement on injury definitions and data collection in studies of football injuries. Injury incidence was reported as the number of injuries per 1000 h of exposure and the number of injuries per squad season. Results: A total of 109 injuries were reported, resulting in a cumulative time-loss of 2536 days. A squad of 25 players sustained 19.7 injuries per season, with an average of 23.3 days (15.7–30.9; 95% CI lower-upper) of absence per injury. Ligament sprains (28%), muscle strains (19%) and physeal injuries (12%) were the most common causes of time-loss. Physeal injuries were the most common severe type of injury (29%), with a mean time-loss of 29.7 days (18.2–41.2; 95% CI lower-upper). Re-injuries accounted for 3% of all injuries and resulted in significantly more time-loss than non-re-injuries (60 vs. 23 days; *p* = 0.01). Conclusion: In the youth academies studied, a team of 25 players sustained an average of 19.7 injuries per season, resulting in a cumulative time-loss of 459 days. Physeal injuries are a major contributor to severe injuries and therefore require special attention.

## 1. Introduction

Elite youth football (soccer) academies play a crucial role in nurturing and developing talented athletes who aspire to reach the highest levels of the sport [1]. However, the pursuit of excellence in football carries inherent risks, including the potential for injury, which can affect both short-term performance and long-term athletic development. In order to effectively prevent sports injuries, Mechelen et al. have developed a widely recognised four-step concept [2]. By examining injury patterns, types, and frequencies, valuable information can be gathered about the potential causes and underlying factors contributing to these injuries.

In the English Premier League, a player suffers an average of 1.3 injuries per season and misses an average of 24 days of training and competition [3]. The data for elite youth athletes are very heterogeneous, with a player suffering between 0.4 and 2.2 injuries per season [4,5]. Re-injury is of particular concern in elite youth football academies due to its severity and potential impact on an athlete’s long-term performance and career prospects [5]. In addition, youth football players have been described as having a higher risk of overuse injuries due to the immaturity of their musculoskeletal system [6,7]. There are some data from English, Dutch, Scandinavian, and Qatari youth academies describing the risk and pattern of injuries in youth football players at the elite club level [8,9,10,11]. There is evidence that the incidence of injury may vary according to the characteristics of the population studied, such as country, age, and level of competition [12,13]. To avoid bias in the analysis of injury data from different highly selective populations of elite youth football players, it is essential that national baseline data are available. For elite youth academies in Germany, no specific data are available to determine the risk of injury and systematically analyse it by type of injury [14].

Therefore, the objectives of this study were to (1) examine the injury pattern and incidence at an elite youth football academy in Germany, (2) monitor overuse injuries over the course of the season, and (3) determine the risk of re-injury in order to develop prevention strategies that will reduce the future injury risk of young players in Germany and thus enable them to achieve optimal career progression.

## 2. Materials and Methods

### 2.1. Study Design and Population

This prospective cohort study was conducted in the 2012/2013 season among male players of an elite youth football academy in Germany, systematically recording injuries in a total of 138 youth players. The study methods and definitions are in accordance with the consensus statement [15]. This study follows the guidelines of the Strengthening the Reporting of Observational Studies in Epidemiology (STROBE) statement.

The youth academy follows a systematic training concept with a sequential modulation of athletic and football specific training. All in all, the younger age groups are trained in a particularly playful manner, while an additional focus is placed on improving strength and athleticism during the transition to the adult level. Depending on the age group, the training load varied from an average of 3 training sessions per week in the U12 and U13 age groups to almost 5 training sessions per week in the U16, U17, and U18 age groups.

Players who joined or left the cohort were included or excluded from the study from their respective dates of entry or exit to ensure that the relevant exposure and injury data were collected and included in the analysis. Each player was tested at the start of pre-season training to establish a baseline. Players with a pre-existing injury at the start of the observation period were included in the study, but the pre-existing injury was not taken into account in the study in accordance with the consensus statement [15]. The study did not document international training and match participation.

### 2.2. Data Collection

Data collection was performed by the team physician responsible for the youth division and the respective medical staff of the youth teams. The training exposure and time-loss injuries of individual players were documented using a standard Injury Record Form [16]. All musculoskeletal injuries were systematically recorded according to the definitions and recommendations for data collection procedures of Fuller et al. [15] (Table 1). A list of injury definitions was attached to the Injury Record Form to increase standardisation between data collectors. The U17 and U19 team coaches electronically recorded exposure data from training and matches, while for the U12 to U16 age groups, the team doctor and medical staff were responsible for recording exposure data. The cause of injury was classified as overuse and trauma-related. However, the exact mechanism of trauma was not specified. Re-injuries are defined as injuries of the same type and at the same site as an index injury that occur after a player returns to full participation from the index injury, according to Fuller et al. [15]. Recurrences are categorised as early, late and delayed recurrences. Non-sport related injuries, such as influenza infections or general illnesses, were excluded from this study. A player was considered injured until he was cleared for full participation in training and matches by the team’s medical staff. Injuries were classified into three severity levels—mild, moderate and severe—based on the number of days the player was unable to participate in training or matches [15]. The injury data was collected with the consent of the players and their guardians.

### 2.3. Data Analysis

Data were recorded and analysed using MS Excel and IBM SPSS Statistics 26. For descriptive analysis, mean and standard deviation were used for parametric data, and median and interquartile range for skewed data. Frequencies were reported as absolute or relative percentages to compare location, type, and severity of injury. The 95% confidence interval indicates the dispersion of the mean time-loss. Injury incidence was calculated as the number of injuries per 1000 h of exposure and the number of injuries per squad season. The number of injuries per season and team was adjusted to a standardised team size of 25 players for better representation. The significance level was set at *p* < 0.05.

## 3. Results

During the observation period, a total of 138 players played in seven teams at the elite football academy studied. The average team size was 19.7 (range 15–22). The cumulative total exposure of all players was 41,973 h, consisting of 36,638 h of training and 5335 h of match play. Anthropometric and player level data are presented in Table 2.

### 3.1. Frequency and Pattern of Injuries

A total of 109 injuries were recorded, of which 72 (66%) occurred during training and 37 (34%) during matches. In a team of 25 players in an elite German youth football academy, 19.8 injuries occur per season. The injuries resulted in a cumulative loss of time of 2536 days, with an average of 23 days per injury.

Of the injuries, 84% involved the lower extremities. The most frequent sites of injury were the ankle, thigh, and knee. Severe injuries mainly affected the knee and foot, while thigh and ankle injuries were mostly mild or moderate in severity (Table 3).

The most common types of injury were ligament sprain, muscle strain, physeal injury, and contusion. The most common subtype was ligament sprain, which accounted for 28.4% of all injuries, mostly affecting the ankle (Table 4). Muscle injuries were almost as common, with muscle strain accounting for 19.3% of all injuries. Overall, muscle injuries—muscle strain and tear—accounted for 25.6% (n = 28) of all injuries. Muscle strains mainly affected the thigh (n = 17, 15.6%), with anterior thigh strains (quadriceps, n = 8, 7.3%) more common than posterior ones (hamstrings, n = 4, 3.7%). In contrast, the hamstring muscles (n = 5, 4.6%) were more frequently affected than the quadriceps (n = 1, 0.9%) in the case of a muscle fibre or bundle tear. In a team of 25 players, approximately 4 strains and 1.5 ruptures can be expected per season.

### 3.2. Injury Severity

Severe injuries (causing >28 days of absence) accounted for 19% (n = 21) of all injuries (Table 5).

The most common types of severe injuries were physeal injuries (n = 6, 29%), fractures (n = 6, 29%), meniscus and cartilage lesions (n = 3, 14%), and muscle fiber or bundle tears (n = 3, 14%). The average (mean) team of 25 players at this level can expect almost four serious injuries per season. On average, each player in the study group missed 18 days per season due to injury. This means that approximately 7% of the season is lost to injury, assuming that the average season lasts 250 days.

**Figure 1:**
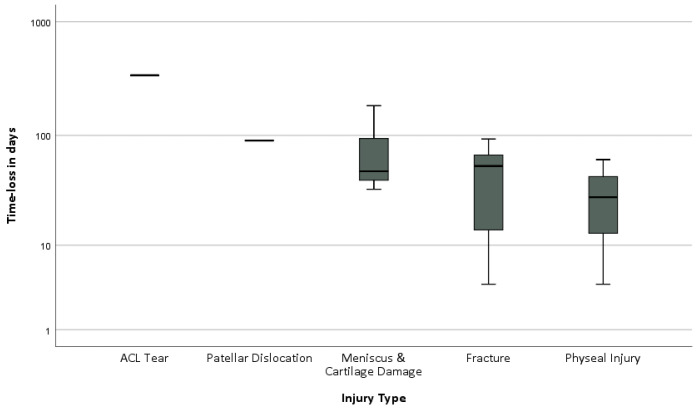
Box plot showing the mean time lost for the most severe injuries.

The most severe injury subtypes were ACL tears, patellar dislocations, meniscus and cartilage injuries, fractures, and physeal injuries, which were associated with longer mean time-loss (Figure 1).

ACL tears, patellar dislocations, and meniscus and cartilage injuries are associated with long time-loss but are relatively rare compared to fractures and physeal injuries. Mean time-loss, incidence per 1000 h of exposure, and incidence per squad season are shown in Table 6. Injuries occurred more frequently in training (66%), with the mean time-loss due to injury being higher in matches (28.9 SD 56.6 vs. 20.4 SD 28.2; *p* = 0.29). However, this difference was not significant. The injury incidence per 1000 h of exposure was 3.4 times higher in competition (6.9 (2.9–11.0) vs. 2.0 (1.5–2.2); *p* < 0.05) than in training.

### 3.3. Variations in Injury Incidence over the Course of a Season

In total, 86 of the 109 time-loss injuries were traumatic. Traumatic injuries accounted for 92% of match injuries and 72% of practice injuries. Overuse injuries accounted for 21% of all injuries.

**Figure 2:**
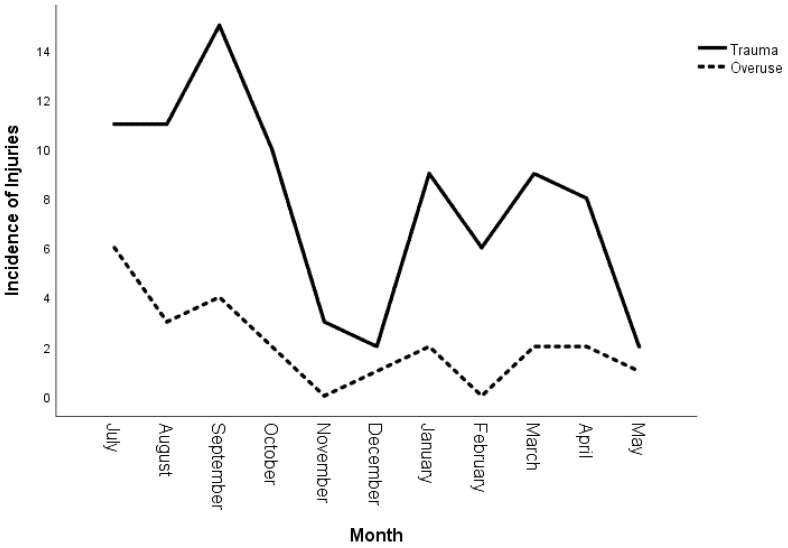
Trauma and overuse injuries throughout the season.

While overuse injuries occurred mainly in the pre-season in July, traumatic injuries occurred primarily in the early championship in September and January (Figure 2).

### 3.4. Re-Injuries

Re-injuries accounted for 3% of all injuries and resulted in significantly more absences than non-re-injuries (60 vs. 23 days; *p* = 0.01). All of the observed recurrences occurred in the time interval from 2 to 12 months after the index injury and can therefore be described as late recurrences.

## 4. Discussion

The impact of injuries in professional and academy football differs significantly. In professional football, injuries are measured in terms of missed competitive matches and their financial impact on player salaries and team performance. However, in academy football, the consequences of injuries must be considered in terms of player development and skill acquisition. On average, each injury excluded players from participating in normal activities for approximately 23 days, with an average of 0.8 injuries per season per player. Consequently, this translates to approximately 7% of the season being lost, which is a significant amount of critical development time. In terms of the development of young players, it therefore makes sense to pay special attention to frequent and severe injuries in prevention programmes.

### 4.1. Frequency and Pattern of Injuries

In the published literature on injury incidence in elite youth football academies, the incidence ranges from 1.3 to 21.1 per 1000 h of exposure, with a mean incidence of time-loss injuries of 5.8 in the U9–U21 age groups [13]. The heterogeneity in the literature may be due to different age structures in the youth sections of different countries, different degrees of professionalisation in youth football, and constitutional differences [17,18]. For example, the youth section of the youth academy examined in this study includes age groups from U12–U19, which is structurally different from professional youth academies in England or Qatar [10,19]. The incidence of injuries in professional youth football is therefore about half that of professional adult football in Europe, as shown by comparisons with data from the Swedish, Norwegian, and English Premier Leagues [20]. The differences with professional football are mainly due to the higher incidence of match-related injuries in adults [21].

In terms of time-loss per injury, Price et al. found an average of just under 22 days in an English youth academy and Materne et al. of 19 days in Qatar [5,19]. The mean incidence of time-loss injuries in the collective studied was 2.6 per 1000 h of exposure (1.7–3.0; 95% CI) or 19.8 team injuries per season, with an average time-loss per injury of 23 days. Accordingly, the incidence of injuries per player or per team over the course of a season and the associated time-loss in the collective studied was at the lower bound but in line with previous findings from youth football academies in other countries [4,5,9,22].

Injuries occurred predominantly in the lower extremity and mainly involved the thigh, knee, and ankle, which is consistent with the findings of previous studies [5,23,24,25]. Ankle and foot injuries accounted for 30% of all injuries. This is consistent with data from a recent review, which found that 79% of all injuries involved the lower limb and between 10 and 38% involved the ankle and foot [13].

In the literature, the incidence of muscle injuries is 27–37% and ligament injuries comprise 15–19% of all injuries in high level youth football [13]. Muscle injuries in male youth football have been shown to be a major cause of injury and downtime in youth academies, predominantly affecting the thigh muscles. The reasons are complex and probably multifactorial, including growth imbalances and developmental differences in the musculature of young people, muscular stiffness, incompliance to prevention programmes, and sports-specific factors such as kicking and stop-and-go movements with frequent changes of direction [26]. The Nordic Hamstring Exercise Programme has been developed to prevent hamstring injuries and several studies have shown its benefits [27]. The benefits of other training programmes, such as plyometric training, are also discussed controversially in the literature [26,28,29]. Despite the existence of effective prevention programmes, the incidence of hamstring injuries in professional and amateur football continues to increase [30]. This may be due to a lack of knowledge about the effectiveness of the programme and a lack of motivation to perform non-specific training [31]. It is necessary to identify the primary extrinsic and intrinsic factors that influence the genesis of thigh muscle injuries before prevention strategies can be further improved. However, due to its limited power, the present study is unable to investigate potential factors influencing muscular injuries in elite youth football.

Contrary to the review by Jones et al., ligament sprains (28.4%) were the primary injury in the study population [13]. The high incidence of ligament sprains in the population studied requires close monitoring by medical staff and may indicate a potential training deficit in proprioception and joint stability.

### 4.2. Injury Severity

The prevalence of serious injuries was 19%, which is comparable to the previous literature findings of 18% [13]. Physeal injuries, which classically occur at the beginning and end of the pubertal growth spurt, were a significant factor in the time-loss of young athletes, accounting for 12% of all injuries and 29% of all severe injuries in the study cohort, with an average incidence of 2.4 per squad season. None of these physeal injuries were associated with any direct trauma. The literature describes growth-related injuries in elite youth football players with lower prevalence and incidence, ranging from 0.8–6 per squad season [4,5,32]. The lower incidence of physeal injuries in previous studies is a possible effect of the increasing professionalisation and intensity of modern elite youth football academies [4,5,9]. This finding is highly relevant as it underlines the absolute necessity of closely monitoring the growth of young athletes in order to identify growth spurts early, adjust training individually, and thus avoid overuse injuries.

Knee injuries, in particular ACL tears, patella dislocations, and meniscus or cartilage damage, were associated with the longest periods of absence, along with fractures. While the severity of the injury means that the injured athlete’s personal development as a player is significantly curtailed, the overall burden on the team is comparable to that of fractures or ligament ruptures due to the low incidence per team season [33]. ACL tears were associated with the longest time-loss among the athletes studied. ACL injuries of the growing skeleton are particularly challenging for the surgeon and are an adverse event to be avoided due to the high risk of recurrence in young athletes [34,35]. The high re-rupture rate of more than 10% after ACL replacement in young athletes justifies the need for innovative prevention programmes, improvements in the surgical procedure and the postoperative rehabilitation protocol with adapted load management, psychological support, and return-to-play tests to allow for a safe return to competition [36,37].

A successfully implemented example of injury prevention in professional football is the FIFA 11+ programme, which is now also used in coach development and recreational sports. 11+ was developed by the FIFA Medical Assessment and Research Centre (F-MARC, Zurich, Switzerland), the Santa Monica Orthopaedic and Sports Medicine Research Foundation (Santa Monica, CA, USA), and the Oslo Sports Trauma Research Centre (Oslo, Norway). The programme aims to improve muscle strength, kinaesthetic sensitivity, and neuromuscular control during static and dynamic movements. The three-part warm-up programme can be completed in under 30 min and, in addition to running and sprinting exercises, includes exercises to strengthen, and improve balance, plyometrics, and agility. In a prospective RCT of 1st and 2nd division college football players, the incidence of injuries of any type was reduced by 46% in the intervention group, with ACL injuries 4.25 times more common in the control group [38].

### 4.3. Variation in Injury Incidence over the Course of a Season

Traumatic injuries were primarily match related, which is in line with previous findings [13]. Overuse injuries, on the other hand, were primarily training related, which explains the finding of an overuse peak incidence during the intensive training months of the pre- and early season from July to September. Traumatic injuries were particularly high in the time periods from August to November and from January to May. This seasonal variability in the incidence pattern of traumatic or overuse injuries is also known from adult professional football. In the UEFA Injury Study, Ekstrand et al. found a peak of overuse injuries in July and an even distribution of traumatic injuries throughout the season [39]. The low incidence of injuries between November and January in the population studied may be explained by the longer winter break in youth football.

### 4.4. Re-Injuries

Injury and inadequate rehabilitation are risk factors for re-injury in professional football [40]. Studies from Scandinavia have shown re-injury rates of between 22 and 30% among professional players [41]. Re-injury rates in youth football in the literature are significantly lower, at 3% in English football academies, which is in line with our findings [5]. Cezarino et al. report a recurrence rate of 9.6% in a Brazilian youth academy [22]. The encouraging, significantly lower rate of re-injury in youth football may be explained by the lower financial pressure on players and clubs, which allows medical staff to make evidence-based decisions in favour of the long-term individual development of the player. The low re-injury rate in European youth football may reflect the good training of coaches in professional youth academies, where individual training guidance has become an important aspect.

### 4.5. Strengths and Limitations

Although the present study is relatively large compared to similarly designed research projects, the power of the study is limited by the cohort size of the 138 athletes studied. A total of 109 injuries were observed, with only the two most common injury types having more than 20 injury cases. According to Bahr and Holme, a study investigating risk factors for injury should observe approximately 20 to 50 injury cases to detect a moderate to strong influence of potential risk factors [42]. To investigate a weak to moderate influence of different factors, approximately 200 subjects are needed. Therefore, potential risk factors for the genesis of different types of injuries could not be investigated because they did not reach the corresponding power calculated by Schmoor et al. [43].

Time-loss of less than 72 h was not recorded in the study population. However, the injury severity classification described by Fuller et al. is based on four categories and, unlike the present study, also includes injuries of less than 72 h. Furthermore, additional training sessions or game exposure as part of courses for the respective junior national teams were not recorded. The inclusion of injuries that prevent normal participation in training and competition for less than 72 h was considered impractical and of little relevance due to the procedures at the football academy.

Another issue could be the temporal context of data collection and the associated lack of timeliness. Given the importance of including recent data in scientific research, several important aspects need to be highlighted in this context. First, there is a lack of prospective injury data in elite youth academies in Germany. In order to fill this gap, these data will provide an essential baseline for comparison with any future dataset, underlining their importance for scientific discourse. In addition, the inclusion of older data opens up the possibility of conducting longitudinal studies [44]. This will allow for the injury history of individual athletes to be tracked and help to understand the long-term effects of injury on their careers.

A strength of our study is the prospective nature of the data, which avoids the potential recall bias seen in self-reported injury studies. According to Hägglund et al., reports from injury surveillance studies covering an entire season provide a reasonable overview of injury patterns and incidence among football players in a given setting [45].

### 4.6. Future Prospects

To further improve the medical management of young players in professional academies, general and specific screening for injury types is needed. Identifying the most common and impactful injuries will allow prevention strategies to be targeted at those that are most detrimental to short-term performance and, more importantly, long-term athletic development. While evidence-based programmes—such as Fifa 11+—that target a wide range of injuries are still considered best practice, therapists may consider tailoring interventions according to age-related patterns [46]. A breakdown of the injury burden by age group, injury type, and diagnosis will provide a better understanding of the impact of injuries and support injury prevention.

## 5. Conclusions

This study provides a detailed characterisation of the injury incidence (2.6 per 100 h or 19.7 per team season) and the average time-loss (23 days) in a highly selective cohort of 138 football players from an elite youth academy in Germany. Physeal injuries are a major contributor to severe injuries and are mainly caused by overuse. They therefore require special attention, effective growth monitoring, and individualised load control for athletes. Overuse injuries tend to occur in the pre-season and may be the result of inadequate training management. Re-injury is less common in elite youth football than in professional football, although it is still associated with prolonged periods of inactivity. The data collected in this study provide valuable insights into injuries in elite youth football in Germany and may contribute to injury prevention efforts.

## Figures and Tables

**Table 1:** Groupings and categories for classifying type of injury [15].

Terminology	Description
Muscle	
Tear	Injuries with structural damage or complete muscle rupture, corresponding to grades IIIa, b and IV according to Müller-Wohlfahrt et al.
Strain	Injuries with functional damage, corresponding to grade II according to Müller-Wohlfahrt et al.
Functional Muscle disorder	Injuries involving functional disorders without structural damage to the musculature (e.g., neuromuscular disorders or fatigue-related muscular disorders)
Bone	
Physeal injury	Physeal injuries other than physeal fractures (e.g., apophysitis)
Mature Bone	
Fracture	All fractures of mature bones caused by trauma
Other Bone injury	Structural bone damage such as bone marrow oedema, stress fractures or periostitis
Ligament	
Sprain/Ligament injury	Strained or torn ligaments responsible for joint stability
Others	
Concussion	Mild traumatic brain injury resulting from head impact or acceleration-deceleration forces
Skin lesion	Mechanical or thermal damage causing a disruption of the skin barrier.
Other injury	Acute functional impairment without diagnosis of specific tissue pathology
Non-specific overuse	Slow-onset functional impairment reported without diagnosis of specific tissue pathology

**Table 2:** Demographic characteristics of the youth academy players.

Age Groups	Total Players	Age (years, SD)	Stature (cm, SD)	Body Mass (kg, SD)	BMI (kg/m^2^, SD)	League
U-12	15	11.8 (0.4)	148.1 (7,1)	42.1 (5.5)	19.2 (1.9)	Kreisklasse A
U-13	18	12.7 (0.5)	156.7 (7.2)	44.7 (6.1)	18.2 (1.8)	Landesliga Rhein-Neckar
U-14	20	13.5 (0.5)	161.4 (7.0)	48.3 (5.9)	18.5 (1.8)	Oberliga Baden-Württemberg
U-15	21	14.6 (0.5)	168.5 (7.3)	55.4 (6.1)	19.5 (2.0)	Regionalliga Süd
U-16	22	15.7 (0.5)	173.8 (6.4)	62.5 (6.2)	20.7 (1.7)	Oberliga Baden-Württemberg
U-17	21	16.6 (0.5)	176.7 (5.2)	69.8 (6.7)	22.4 (1.6)	Bundesliga
U-19	21	18.0 (0.8)	181.7 (7.8)	75.1 (7.5)	22.7 (2.1)	Bundesliga
All Teams	138	14.9 (2.1)	166.7 (6.9)	56.8 (6.3)	20.4 (1.8)	

**Table 3:** Injury frequency and severity by body region.

Injury Location	Frequency n (%)	4–7 Days	8–28 Days	>28 Days
Head/Neck	5 (5)	2 (4)	3 (7)	-
Shoulder/Clavicula	2 (2)	2 (4)	-	-
Ellbow	1 (1)	-	1 (2)	-
Forearm	3 (3)	1 (2)	1 (2)	1 (5)
Hand	4 (4)	3 (7)	-	1 (5)
Trunk	3 (3)	1 (2)	2 (5)	-
Hip/Pelvis	5 (5)	-	2 (5)	3 (14)
Thigh	26 (24)	15 (33)	9 (21)	2 (10)
Knee	21 (19)	5 (11)	10 (23)	6 (29)
Lower leg/Calf	6 (6)	3 (7)	3 (7)	-
Ankle	22 (20)	9 (20)	11 (26)	2 (10)
Foot/Toe	11 (10)	4 (9)	1 (2)	6 (29)
Overall	109 (100%)	45	43	21

**Table 4:** Injury frequency and time-loss by region and subtype of injury.

Diagnosis	Frequency n (%)	Total Time-Loss (Days)	Median Time-Loss (IQ 25th–75th)
Head and neck	5 (4.6%)	44	10 (4.5–12.5)
Concussion	2 (1.8%)	21	10.5 (10–10.5)
Nasal bone fracture	2 (1.8%)	18	9 (4–9)
Cut	1 (0.9%)	5	5 (5–5)
Upper Limb	10 (9.2%)	225	7 (4.75–31.75)
AC-Contusion	1 (0.9%)	4	4 (4–4)
Shoulder Contusion	1 (0.9%)	5	5 (5–5)
Ellbow Contusion	1 (0.9%)	8	8 (8–8)
Forearm Contusion	1 (0.9%)	7	7 (7–7)
Forearm Fracture	2 (1.8%)	91	45.5 (45–46)
Hand Contusion	2 (1.8%)	11	5.5 (5–6)
Hand/Finger sprain	1 (0.9%)	6	6 (6–6)
Hand/Finger fracture	1 (0.9%)	93	93 (93–93)
Trunk	3 (2.8%)	42	11 (5.0–11)
Overuse unspecific pathology	2 (1.8%)	37	18.5 (11–18.5)
Functional muscle disorder	1 (0.9%)	5	5 (5–5)
Hip and Pelvis	5 (4.6%)	176	36 (13.5–58.5)
Physeal injury (avulsion)	2 (1.8%)	68	34 (12–34)
Physeal injury (apophysitis)	2 (1.8%)	97	48.5 (36–48.5)
Gluteal strain	1 (0.9%)	15	15 (15–15)
Thigh	26 (23.9%)	317	6.0 (4–32.5)
Torn Hamstrings	5 (4.6%)	140	23.0 (13–44)
Torn Quadriceps	1 (0.9%)	15	15 (15–15)
Thigh Contusion	3 (2.8%)	15	5 (4–5)
Adductor strain	5 (4.6%)	56	11 (5.0–17.5)
Hamstring strain	4 (3.7%)	17	4 (4–4.75)
Quadrizeps strain	8 (7.3%)	74	6 (4–13)
Knee	21 (19.3%)	929	14 (7.5–38)
Knee Sprain/Ligament	9 (8.3%)	103	10 (4–16)
Meniscus and Cartilage Damage	3 (2.8%)	264	48 (33–48)
Physeal injury (apophysitis)	7 (6.4%)	133	14 (8–28)
Patellar dislocation	1 (0.9%)	90	90 (90–90)
Torn ACL	1 (0.9%)	339	339 (339–339)
Lower leg and calf	6 (5.5%)	47	7.5 (5.75–9.75)
Functional muscle disorder	1 (0.9%)	12	12 (12–12)
Torn Calf muscle	1 (0.9%)	8	8 (8–8)
Calf muscles strain	3 (2.7%)	20	6 (5–6)
Calf Cut	1 (0.9%)	7	7 (7–7)
Foot and Ankle	33 (30.3%)	752	14 (5–31)
Ankle Sprain/Ligament	19 (17.4%)	295	13.0 (7–19)
Ankle contusion	1 (0.9%)	4	4 (4–4)
Ankle fracture	1 (0.9%)	57	57 (57–57)
Ankle Stress injury	1 (0.9%)	5	5 (5–5)
Physeal injury (apophysitis)	2 (1.8%)	88	44 (39–44)
Foot/Toes fracture	4 (3.7%)	185	56 (17–65.8)
Foot Contusion	3 (2.8%)	96	4 (4–4)
Foot sprain	2(1.8%)	22	11 (4–11)
			Mean (SD)
Total	109 (100%)	2536	23.3 (40.1)

**Table 5:** Injury frequency and severity by type of injury.

Injury Type	Total n (%)	4–7 Days n (%)	8–28 Days n (%)	>28 Days n (%)
Ligament sprain	31 (28)	13 (29)	17 (40)	1 (5)
Muscle strain	21 (19)	14 (31)	7 (16)	-
Physeal injury	13 (12)	1 (2)	6 (14)	6 (29)
Contusion	13 (12)	11 (24)	1 (2)	1 (5)
Fracture	10 (9)	2 (4)	2 (5)	6 (29)
Muscle tear	7 (6)	-	5 (12)	2 (10)
Meniscus and Cartilage lesion	3 (3)	-	-	3 (14)
Functional muscle disorder	2 (2)	1 (2)	1 (2)	-
Concussion	2 (2)	-	2 (5)	-
Cut	2 (2)	2 (4)	-	-
ACL tear	1 (1)	-	-	1 (5)
Dislocation	1 (1)	-	-	1 (5)
Stress injury	1 (1)	1 (2)	-	-
Other overuse	2 (2)	-	2 (5)	-

**Table 6:** Characteristics grouped by type and ranked by severity of injury.

Injury Type	Incidence n (%)	Incidence/1000 h	Incidence/Squad-Season *	Incidence/Squad-Season **	Mean Time-Loss (95%-KI)	Training/Match-Ratio
Ligament sprain	31 (28)	0.7	4.4	5.6	13.8 (8.9–18.6)	1.6
Muscle strain	21 (19)	0.5	3.0	3.8	8.7 (5.6–11.7)	6
Physeal injury	13 (12)	0.3	1.9	2.4	29.7 (18.2–41.2)	5.5
Contusion	13 (12)	0.3	1.9	2.4	11.5 (0–25.5)	1.2
Fracture	10 (9)	0.2	1.4	1.8	44.4 (21.9–66.9)	0.67
Torn Muscle	7 (6)	0.2	1.0	1.3	16 (13–43)	0.75
Meniscus and Cartilage	3 (3)	0.1	0.4	0.6	88 (0–293)	-
Functional muscle disorder	2 (2)	0.1	0.3	0.4	8.5 (0–53.0)	1
Concussion	2 (2)	0.1	0.3	0.4	10.5 (4.2–16.9)	1
Cut	2 (2)	0.1	0.3	0.4	6 (0–18.7)	1
ACL tear	1 (1)	0.05	0.1	0.2	339	-
Dislocation	1 (1)	0.05	0.1	0.2	90	-
Stress injury	1 (1)	0.05	0.1	0.2	5	-
Other overuse	2 (2)	0.1	0.3	0.4	18.5 (0–113.8)	-
Overall	109 (100)	2.6	15.6	19.7	23.3 (15.7–30.9)	1.95

Calculated based on * mean team size ** team size of 25 players.

## Data Availability

The data presented in this study are available on request from the corresponding author. The disclosure of sensitive data of professional footballers may be prohibited by the participating club.
